# Large language models in healthcare: applications, evaluation frameworks, and governance pathways — a scoping review and multidimensional framework

**DOI:** 10.3389/fdgth.2026.1865568

**Published:** 2026-09-18

**Authors:** Joao C. Ferreira, Isabel Rosa

**Affiliations:** 1 Faculty of Logistics, Molde University College, Molde, Norway; 2 Inov Inesc, Lisbon, Portugal; 3 IISCTE – IUL (Instituto Universitário de Lisboa), Lisbon, Portugal; 4 Genesis Digital Solutions, Lisbon, Portugal

**Keywords:** algorithmic bias, clinical decision support, clinical documentation, digital health, evaluation framework, generative artificial intelligence, governance, large language models

## Abstract

**Background:**

Large language models (LLMs) are increasingly evaluated for healthcare applications spanning clinical documentation, decision support, patient communication, research assistance, and operational workflows. Despite rapid adoption interest, evidence remains heterogeneous and standardised approaches for evaluation and governance are not yet consistently applied.

**Objective:**

To map healthcare applications of LLMs, synthesise reported outcomes and risks, and propose a multidimensional evaluation and governance framework oriented toward digital-health implementation.

**Methods:**

A scoping review was conducted following the methodological guidance of Arksey and O'Malley, Levac et al., and the Joanna Briggs Institute, with reporting aligned to the PRISMA-ScR checklist. The protocol was prospectively registered on the Open Science Framework https://doi.org/10.17605/OSF.IO/SWP78). Searches were performed in PubMed/MEDLINE, Embase, Scopus, Web of Science, IEEE Xplore, ACM Digital Library, and the ACL Anthology, supplemented by medRxiv/bioRxiv preprint searches and grey-literature scanning of WHO, FDA, and EU AI-Office guidance, covering 1 January 2019–30 April 2026. Title/abstract and full-text screening were carried out in duplicate; inter-rater agreement was Cohen's κ = 0.78. Data were extracted in duplicate using a piloted form. Synthesis followed Braun and Clarke's six-phase reflexive thematic analysis. The complete list of 78 included studies is provided in Supplementary File S4.

**Results:**

Seventy-eight studies met inclusion criteria; 68% were published between 2023 and 2025. Five application domains were identified: (1) clinical documentation and summarisation; (2) clinical decision support and reasoning assistance; (3) patient communication and health-literacy support; (4) biomedical research and knowledge synthesis; and (5) administrative and operational use cases. The evidence base was dominated by benchmark and simulated-workflow studies (89%), with limited prospective workflow-embedded evaluations (11%). Reported benefits concentrated on documentation efficiency, text quality, and knowledge synthesis; safety-relevant risks included hallucinated content, omission of clinically critical information, demographic bias, privacy vulnerabilities, limited explainability, and automation bias. Studies were geographically concentrated in North America and East Asia, with limited representation from Sub-Saharan Africa, South Asia, and Latin America.

**Conclusions:**

Current evidence supports cautious deployment of LLMs in selected healthcare tasks under structured oversight. Translational progress depends on prospective evaluation, standardised reporting, equity-focused audits, and lifecycle governance with continuous monitoring. The proposed five-dimensional framework (technical performance, clinical validity, equity, workflow integration, governance) coupled with a three-tier risk model is intended to support researchers and healthcare organisations in assessing readiness and implementing LLM-enabled tools responsibly.

## 1 Introduction

Healthcare systems are undergoing rapid digital transformation driven by the increasing availability of electronic health records (EHRs), patient-generated data, biomedical literature, and multimodal clinical information. Large language models (LLMs), built on transformer architectures and trained on large-scale textual corpora, have emerged as a class of artificial intelligence (AI) systems capable of natural language understanding, generation, summarisation, and reasoning (1–3). Their introduction into healthcare settings has catalysed interest in clinical decision support, medical documentation, patient engagement, and biomedical research workflows (75, 77).

Unlike earlier rule-based or narrowly supervised natural language processing systems, LLMs demonstrate capabilities across diverse tasks with limited task-specific retraining (3, 4). In healthcare, these capabilities include drafting clinical notes, summarising patient histories, assisting with differential-diagnosis generation, supporting patient communication, and synthesising biomedical literature. At the same time, their probabilistic nature introduces concerns related to factual reliability and hallucination, bias propagation, limited interpretability, automation bias, and accountability (5–7).

For the purposes of this review, LLMs are defined as transformer-based deep-learning models trained on large corpora to perform language understanding and generation tasks (1–3). This includes general-purpose foundation models as well as medically adapted or domain-specialised variants. Multimodal extensions integrating imaging, structured data, or omics data are considered when language processing remains a core component of system functionality.

While early demonstrations highlight promising performance across question answering, summarisation, and documentation tasks, healthcare deployment requires more than benchmark accuracy. Clinical environments impose constraints related to patient safety, regulatory compliance, data protection, workflow integration, and medico-legal responsibility (7). Models that perform well in experimental conditions may fail under real-world variability, diverse patient populations, or incomplete data contexts. Common natural language generation metrics such as BLEU or ROUGE inadequately capture clinical correctness, contextual appropriateness, or potential harm; healthcare-specific evaluation must incorporate factuality, safety risk, bias assessment, robustness across subpopulations, interpretability, and post-deployment monitoring (7, 8).

Existing reviews (9–14, 79) focus predominantly on performance benchmarks or surface application examples, without systematically connecting applications, measurable outcomes, evaluation methodologies, and governance implications. Few studies articulate minimum evaluation standards required prior to clinical deployment or propose structured monitoring strategies after implementation (15–17).

Aim and research questions. This scoping review aims to bridge the gap between technical capability and responsible healthcare deployment by addressing five research questions: RQ1. What healthcare application domains for LLMs are described in the peer-reviewed literature, and how can they be taxonomically organised? RQ2. What measurable outcomes (task correctness, efficiency, user experience, safety, equity) are reported, and how are they defined? RQ3. What evaluation methodologies are currently used, and what are their methodological limitations? RQ4. What risks, ethical concerns, and governance gaps are identified, and which mitigation strategies are proposed? RQ5. What multidimensional evaluation and governance framework can support responsible LLM deployment within digital-health ecosystems? The contribution of this review is therefore both descriptive — characterising the current evidence base — and normative: proposing a five-dimensional, risk-tiered evaluation and governance framework intended to be operationalised by researchers and healthcare organisations.

## 2 Methods

### 2.1 Protocol and registration

The review protocol was prospectively registered on the Open Science Framework. The OSF repository contains: search strategies per database with exact execution dates; deduplication procedure; extraction spreadsheet; and the complete list of 78 included studies (Supplementary File S4). Any deviations from the registered protocol are documented in §2.10 (Deviations from protocol) and in the OSF record.

### 2.2 Reporting standards

The review was conducted following the methodological framework originally outlined by Arksey and O'Malley (18) and refined by Levac et al. (19), and incorporates updated guidance from the Joanna Briggs Institute (JBI) for scoping reviews (20). Reporting follows the Preferred Reporting Items for Systematic Reviews and Meta-Analyses extension for Scoping Reviews (PRISMA-ScR) (21); the completed PRISMA-ScR checklist is provided as Supplementary File S1. A scoping design was selected because the field is rapidly evolving, study designs are heterogeneous, and the objective is conceptual mapping rather than pooled effect estimation.

### 2.3 Eligibility criteria

Studies were eligible if they (i) reported on the development, evaluation, implementation, or analysis of LLMs in healthcare contexts; (ii) presented empirical results, systematic analysis, or structured conceptual frameworks; (iii) were published as peer-reviewed journal articles, conference proceedings, or refereed preprints with downstream peer-reviewed publication; and (iv) were available in English, Portuguese, or Spanish (a pragmatic restriction reflecting the language competencies of the review team).

Studies were excluded if they (i) addressed exclusively non-healthcare domains; (ii) reported technical model architectures without any healthcare-relevant evaluation; or (iii) were editorials, opinion pieces, or commentaries lacking analytical or empirical contribution.

The language restriction to English, Portuguese, and Spanish is acknowledged as a pragmatic limitation reflecting team competencies. Studies published in Mandarin, Korean, Arabic, or other non-Latin-script languages may have been excluded, potentially underrepresenting research from East Asian, Middle Eastern, and South Asian institutions (see also §6.2).

### 2.4 Information sources and search strategy

We searched seven bibliographic databases — PubMed/MEDLINE, Embase, Scopus, Web of Science Core Collection, IEEE Xplore, the ACM Digital Library, and the ACL Anthology — supplemented by medRxiv and bioRxiv preprint servers and a structured grey-literature scan covering the World Health Organization, the U.S. Food and Drug Administration, Health Canada, the U.K. Medicines and Healthcare products Regulatory Agency, and the EU AI-Office guidance pages. The search covered publications from 1 January 2019 to 30 April 2026; the start date reflects the emergence of large-scale transformer-based language models (1–3). In-press records identified through database alerts and citation chasing during the writing phase were retained where the publishing journal had assigned a DOI.

The core search query, applied with database-appropriate field tags, was: (“large language model*” OR “LLM” OR “GPT-3” OR “GPT-4” OR “ChatGPT” OR “foundation model*” OR “generative AI”) AND (“healthcare” OR “clinical” OR “medicine” OR “hospital” OR “patient”) AND (“evaluation” OR “validation” OR “assessment” OR “implementation” OR “governance” OR “safety” OR “bias” OR “fairness”).

The search strategy was peer-reviewed by a health-sciences information specialist using the PRESS 2015 checklist before execution (22). Full search histories as executed (including all MeSH and Emtree terms added following PRESS review) were exported directly from each database immediately after execution. Full search strings for each database, the PRESS feedback log, and the dates of search execution are provided in Supplementary File S2. The search strings in Supplementary File S2 represent the final executed versions incorporating all modifications recommended during PRESS peer review. Backward and forward citation chasing of all included studies was conducted using Web of Science and Google Scholar (1–6 April 2026). Note: Supplementary File S2 previously recorded these dates as 1–6 May 2026; this was a clerical error and has been corrected in the revised S2.

### 2.5 Study selection

Records were imported into Covidence systematic-review software, deduplicated, and screened in two stages. A pilot screening exercise on 100 randomly selected records was performed jointly by both reviewers to refine the eligibility criteria. Title/abstract screening was then conducted in duplicate by both authors working independently; inter-rater agreement at this stage was Cohen's κ = 0.78 (95% CI 0.74–0.82), *n* = 5,520 records, indicating substantial agreement. Full-text screening was likewise performed in duplicate, with disagreements resolved by discussion and, where necessary, third-party adjudication by an external clinical informatician; inter-rater agreement at full-text stage was Cohen's κ = 0.83 (95% CI 0.78–0.88), *n* = 366 reports. The PRISMA-ScR flow diagram is presented in Figure 1.

**Figure 1:**
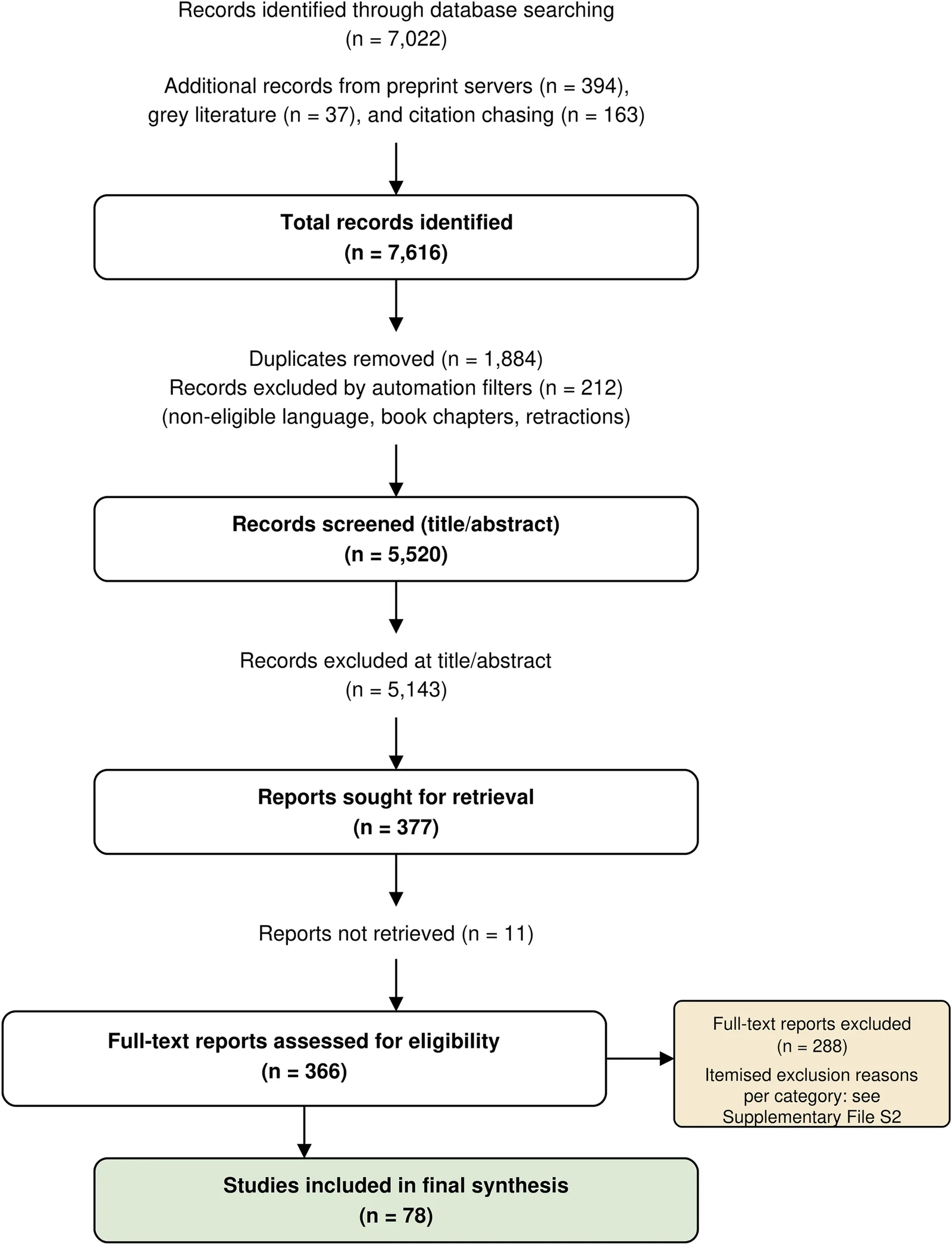
PRISMA-ScR flow diagram of study selection. Numbers in each stage (records identified, deduplicated, screened, full-text assessed, and included) are reported in Supplementary File S2.

### 2.6 Data extraction

A data-extraction form was developed iteratively, piloted on ten included studies, and refined before extraction of the full sample. Two reviewers extracted data independently and reconciled discrepancies by consensus. Extracted variables included: bibliographic information (authors, year, country, journal); population and clinical setting; model family, version, and access mode (proprietary API, open weights, fine-tuned); deployment setting (benchmark, simulated workflow, real-world clinical integration); evaluation design; primary and secondary outcome categories; reported risks and mitigation strategies; and governance reporting (bias analysis, privacy/data handling, human oversight, post-deployment monitoring). The full extraction template and the codebook are available as Supplementary File S3.

The complete list of included studies with study-level extraction data is provided in Supplementary File S4.

### 2.7 Critical appraisal

Consistent with current JBI guidance for scoping reviews (20), formal critical appraisal of individual studies was not undertaken because the purpose of this review is conceptual mapping rather than effect estimation or evidence-strength quantification. To allow readers to gauge evidence maturity, however, four methodological-quality flags were captured during extraction and are reported in Table 1: deployment setting, evaluation design, equity reporting, and oversight reporting.

**Table 1:** Characteristics of included studies (*n* = 78).

Characteristic	Category	*n* (%)
Clinical Domain		
	Clinical documentation & summarisation	21 (27%)
	Clinical decision support & reasoning assistance	27 (35%)
	Patient communication & health-literacy support	20 (26%)
	Biomedical research & knowledge synthesis	6 (8%)
	Administrative & operational use cases	4 (5%)
Deployment Setting		
	Benchmark/retrospective only	52 (67%)
	Simulated workflow	17 (22%)
	Real-world clinical integration	9 (11%)
Model Type		
	Proprietary API (e.g., GPT-4 class)	49 (63%)
	Open-source base models	12 (15%)
	Fine-tuned domain models	14 (18%)
	Multimodal LLMs	3 (4%)
Evaluation Design		
	Cross-sectional benchmarking	41 (53%)
	Retrospective chart comparison	21 (27%)
	Prospective observational	10 (13%)
	Controlled trial	6 (8%)
Governance Reporting		
	Bias analysis reported	18 (23%)
	Privacy/data handling described	26 (33%)
	Human oversight specified	44 (56%)
	Post-deployment monitoring described	9 (11%)

### 2.8 Synthesis

Given heterogeneity of study designs and outcomes, synthesis followed Braun and Clarke's six-phase reflexive thematic-analysis approach (23): (i) familiarisation with the corpus; (ii) generation of initial codes; (iii) construction of candidate themes; (iv) reviewing themes against extracted data; (v) defining and naming themes; and (vi) writing up. A codebook was prepared collaboratively and revised through weekly consensus meetings. Thematic saturation was assessed informally; new themes ceased to emerge after analysis of approximately 60 studies.

### 2.9 Reflexivity and positionality

The review team comprises researchers in digital health, applied AI, and clinical informatics; one author has clinical-research collaboration experience and the other a logistics and information-systems background. Neither author is employed by, holds equity in, nor has received personal funding from a commercial LLM vendor. The framework developed in §5 reflects this perspective; alternative framings (for example, a clinician-only or patient-only standpoint) might emphasise different evaluative priorities.

### 2.10 Deviations from protocol

Two deviations from the registered protocol are noted. First, the search end-date was extended from December 2025 to 30 April 2026 to capture rapidly emerging 2026 publications, including several papers in press at the time of registration. Second, three Spanish-language records were retained in full-text screening to reflect the team's language competencies; none ultimately met the inclusion criteria.

## 3 Results

### 3.1 Study selection

Searches of seven bibliographic databases yielded 7,022 records; preprint servers (medRxiv, bioRxiv) contributed 394 records; grey-literature scanning contributed 37 records; and forward/backward citation chasing of all included full-texts (conducted 1–6 April 2026 via Web of Science and Google Scholar) contributed 163 additional candidates, for a total of 7,616 records before deduplication. Following removal of 1,884 duplicates and 212 records flagged by automation filters (non-eligible languages, book chapters, retractions), 5,520 records were screened at title/abstract. Of these, 5,143 were excluded, leaving 377 reports sought for retrieval; 11 were not retrieved; and 366 reports were assessed for full-text eligibility. Following full-text review, 78 studies met the inclusion criteria. The PRISMA-ScR flow diagram (Figure 1) has been updated to reflect these complete numbers (see also Supplementary File S2). Publication volume increased sharply after 2022, with 68% of included studies published between 2023 and 2025 and a further 14% appearing in 2026 (in press at the time of writing).

### 3.2 Characteristics of included studies

Table 1 summarises study characteristics. 63% (*n* = 49) of studies evaluated proprietary GPT-class APIs, 18% (*n* = 14) used domain-fine-tuned models, 15% (*n* = 12) used open-source base models, and 4% (*n* = 3) evaluated multimodal LLMs. 67% (*n* = 52) reported benchmark or retrospective designs only, 22% (*n* = 17) described simulated-workflow studies, and 11% (*n* = 9) described real-world clinical integration. Bias analyses were reported in 23% (*n* = 18) of studies, privacy/data-handling considerations in 33% (*n* = 26), and post-deployment monitoring in 11% (*n* = 9). Geographically, 56% (*n* = 44) of studies originated from North American institutions, 22% (*n* = 17) from East Asia, 15% (*n* = 12) from Europe, and 7% (*n* = 5) from other regions; Sub-Saharan Africa, South Asia, and Latin America were each represented by fewer than three studies. This distribution is itself an equity finding (§4.6) and limits external validity for low-resource settings.

### 3.3 Application domains

LLM applications in healthcare clustered into five domains: (1) clinical documentation and summarisation; (2) clinical decision support and reasoning assistance; (3) patient communication and health-literacy support; (4) biomedical research support; and (5) administrative and operational optimisation. Each is summarised below; representative references are tabulated thematically in Tables 2–8.

**Table 2:** Human-Centered & holistic evaluation frameworks.

Ref	Lead Author	Year	Major Contribution
(24)	Tam TYC	2024	Derived QUEST framework for human evaluation of healthcare LLMs (3-phase workflow: Planning, Implementation/Adjudication, Scoring/Review; covers dimensions, evaluators, metrics, statistics)
(25)	Hong C	2025	Applied unified evaluation framework to real In-Basket patient messages in Epic; bridged clinician qualitative feedback with automated quantitative metrics (clarity, completeness, safety)
(26)	Seo J	2024	Developed & validated usability-tested evaluation framework specifically for LLM-generated emergency department medical documentation
(27)	Bedi S	2026	Introduced MedHELM—clinician-validated holistic taxonomy + benchmark covering 5 real-world medical task categories (Nature Medicine)
(28)	Livingston L	2025	Clinician-in-the-loop reproducible evaluation method for generative AI in healthcare
(29)	He YV	2025	ASTRID—automated, scalable triad framework for evaluating RAG-based clinical question-answering systems
(30)	Chang Q	2025	2025 expert consensus guidelines for retrospective evaluation of LLM applications in clinical scenarios
(31)	Croxford E	2024	Human evaluation framework correlated with automated metrics for natural language generation of medical diagnoses
(32)	Reddy S	2024	Role of explainable AI + evaluation frameworks for safe & effective LLM integration in healthcare

For scoping & systematic reviews of applications, utility & evaluations, see Table 3.

**Table 3:** Scoping & systematic reviews of applications, utility & evaluations.

Ref	Lead Author	Year	Major Contribution
(9)	Park Y-J	2024	Scoping review mapping research landscape and clinical utility of LLMs in medicine
(10)	Zhang Z	2026	Scoping review of LLM applications, future directions, and implementation in healthcare
(11)	Shool S	2025	Systematic review of LLM evaluation methods specifically in clinical medicine
(12)	Artsi Y	2025	Systematic review of LLMs in real-world clinical workflows and implementation barriers
(13)	Wang L	2024	Systematic review of applications and concerns of conversational LLMs (ChatGPT etc.) in healthcare
(14)	Neveditsin N	2025	Comprehensive clinical insights review of language models in medicine
(33)	Ullah E	2024	Scoping review of LLM challenges/barriers in diagnostic medicine with focus on digital pathology
(34)	Woo B.F.Y.	2026	Scoping review of LLMs for clinical documentation

For bias, fairness & demographic disparities, see Table 4.

**Table 4:** Bias, fairness & demographic disparities.

Ref	Lead Author	Year	Major Contribution
(35)	Yang Y	2024	Unmasked & quantified racial bias of LLMs specifically in medical report generation
(36)	Omar M	2025	Systematic review evaluating & addressing demographic disparities in medical LLMs
(37)	Benkirane K	2025	Practical approaches to diagnose and treat bias in LLMs for clinical decision-making
(38)	Rani M	2024	Comprehensive overview of biases, trustworthiness challenges, and mitigation strategies in healthcare LLMs
(39)	Mirza V	2025	Comparative analysis of gender, racial, and age biases across occupational and crime scenarios
(40)	Zhou Y	2025	Performance challenges of LLMs in low-resource healthcare settings from a demographic fairness lens
(41)	Zhao Y	2024	Explored whether LLMs can replace doctors, focusing on bias in disease diagnosis

For hallucination assessment & benchmarks, see Table 5.

**Table 5:** Hallucination assessment & benchmarks.

Ref	Lead Author	Year	Major Contribution
(42)	Gu C	2024	LENS—multi-layer evaluation framework for hallucination in generative AI systems
(43)	Salehi S	2025	Opportunities and hallucination challenges of agentic AI & LLMs in radiology
(44)	Zuo K	2025	MedHallBench—dedicated new benchmark for assessing hallucination in medical LLMs

For ethics, safety, guardrails & harm reduction, see Table 6.

**Table 6:** Ethics, safety, guardrails & harm reduction.

Ref	Lead Author	Year	Major Contribution
(45)	Fareed M	2025	Systematic review of ethical considerations of LLMs in healthcare and medicine
(46)	Haltaufderheide J	2024	Systematic review on ethics of ChatGPT and other LLMs in medicine/healthcare
(47)	Hakim JB	2025	Highlighted need for guardrails of LLMs in pharmacovigilance and other safety-critical medical settings
(48)	Moell B	2025	Harm-reduction strategies and perspectives for patients/clinicians using LLMs in medicine

For clinical performance, dialogue, documentation & real-world applications, see Table 7.

**Table 7:** Clinical performance, dialogue, documentation & real-world applications.

Ref	Lead Author	Year	Major Contribution
(49)	Waldock WJ	2024	Systematic review & meta-analysis of accuracy/capability of AI solutions in health care examinations & certificates
(50)	Fraile Navarro D	2025	Expert (human) evaluation of LLMs for clinical dialogue summarization
(51)	Wu J	2025	Clinical pathway-aware LLMs for reliable & transparent medical dialogue
(52)	Li Y	2026	Evaluation of LLMs for real-world perioperative clinical consultation
(53)	Rocha H	2026	Direct comparison of foundation models vs physicians on textual & multimodal ophthalmological questions

For foundation models, multimodal LLMS & radiology/imaging, see Table 8.

**Table 8:** Foundation models, multimodal LLMs & radiology/imaging.

Ref	Lead Author	Year	Major Contribution
(54)	Paschali M	2025	Comprehensive review: Foundation models in radiology—what, how, why, and why not (Radiology)
(55)	Wong IN	2024	Leveraging foundation models and LLMs in medical artificial intelligence
(56)	Nam Y	2025	Current state and future directions of multimodal LLMs in medical imaging
(57)	Gupta A	2026	Primer on foundation models in radiology tailored for pediatric radiologists
(58)	Wang J	2026	Empowering liver cancer diagnosis/treatment using foundation models

#### 3.3.1 Clinical documentation and summarisation

LLMs are commonly evaluated for generating or summarising clinical documentation, including discharge summaries, progress notes, and structured extraction from free-text EHR narratives (59, 60). Reported benefits relate to drafting efficiency and text quality; risks include factual drift and omission errors, motivating clinician-in-the-loop validation (5, 6, 34, 60). Recent scoping work specifically on LLM-generated clinical documentation highlights persistent gaps in prospective evaluation (34).

#### 3.3.2 Clinical decision support and reasoning assistance

LLMs have been evaluated for differential-diagnosis generation, triage suggestion, and clinical question answering. Benchmark studies report substantial performance on examination-style questions (49, 61, 62, 73, 74); however, safety-relevant failure modes include hallucinated references and overconfident incorrect reasoning (5, 6, 43, 44). Retrieval-augmented generation (RAG) has been explored to ground outputs in external evidence sources (29, 63); clinical-pathway-aware decoding approaches have been proposed for transparency (51).

#### 3.3.3 Patient communication and health-literacy support

LLMs have been applied to draft patient messages and simplify complex medical language (25, 64). Comparative studies report higher-rated empathy and quality for AI-drafted responses in specific settings, although clinical appropriateness and accountability remain concerns (64). Bias may emerge as differential tone or content across demographic contexts, reinforcing the need for equity-focused audits (7, 35, 36, 41, 65).

#### 3.3.4 Biomedical research and knowledge synthesis

LLMs can support literature screening and summarisation, but risks include fabricated citations and inaccurate synthesis; verification workflows remain essential (66, 67).

#### 3.3.5 Administrative and operational optimisation

Administrative applications include coding assistance, billing documentation support, and operational triage (68, 72). These may represent lower-risk deployment candidates, although longitudinal impact and cost-effectiveness data remain limited.

## 4 Reported outcomes and measured impact

Published evaluations of LLMs in healthcare reported outcomes that fell into four categories: (1) task performance and correctness; (2) efficiency and workflow effects; (3) user experience and acceptability; and (4) safety, equity, and risk-related outcomes (Figure 2). Across categories the evidence base was dominated by retrospective studies, controlled benchmarks, and simulated workflow tasks, with relatively few prospective evaluations embedded in routine care.

**Figure 2:**
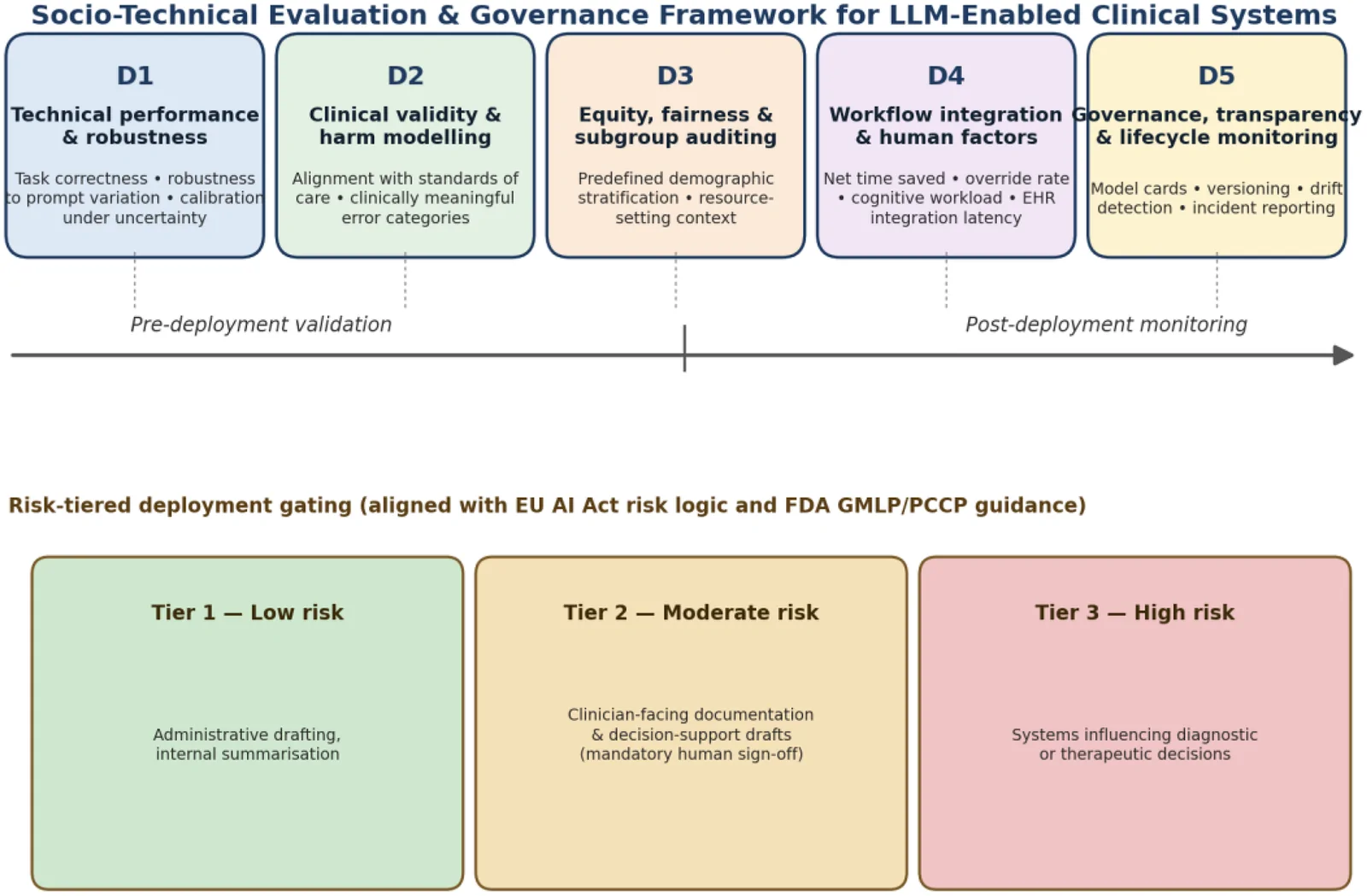
Socio-technical evaluation framework for LLM-enabled clinical systems.

### 4.1 Task performance and correctness

Task performance was most commonly reported using benchmark-style accuracy metrics (e.g., multiple-choice correctness, agreement with reference answers, or rubric-based scoring). Studies evaluating medical knowledge and reasoning report that LLMs can achieve high scores on examination-style questions under controlled conditions (49, 61, 62). However, benchmark scores provide limited information about calibration under uncertainty and performance in the presence of incomplete, noisy, or contradictory clinical data — conditions that are common in real-world settings.

For text-generation tasks (e.g., summarisation, note drafting), performance was frequently assessed using expert review (31, 50) and, less frequently, automated overlap metrics such as ROUGE or BERTScore (8, 26). Domain experts typically judged factual consistency, completeness, and clinical relevance. The literature suggests that aggregate text-similarity metrics alone are poorly aligned with clinically meaningful errors (e.g., omission of contraindications or misattribution of diagnoses) and should not be used as a sole indicator of quality (8, 31).

Calibration — the alignment between expressed confidence and empirical correctness — was reported in only a minority of studies despite its centrality to safe deployment (6, 44). Where examined, LLMs often exhibited overconfidence in factually incorrect outputs, particularly in low-resource medical specialties (40). Abstention behaviour (the model declining to answer when uncertain) was rarely measured, although it is increasingly recognised as a desirable property in clinical contexts (32, 47).

### 4.2 Factuality, hallucination, and error modes

A recurring finding was the coexistence of fluent outputs with clinically consequential errors, including hallucinated facts or references and plausible-but-incorrect clinical statements (5, 6, 43). A taxonomy of hallucination types — adapted from Ji et al. (6) and MedHallBench (44) — distinguishes (i) intrinsic hallucinations, (ii) extrinsic hallucinations, (iii) misattribution, and (iv) clinically consequential omission. In healthcare contexts, omission of safety-critical information (e.g., a contraindication) may be as harmful as fabrication. Retrieval-augmented generation (RAG) was the most frequently proposed mitigation (29, 63); however, RAG itself introduces failure modes when retrieval sources are incomplete, outdated, or irrelevant (29).

### 4.3 Efficiency and workflow impact

Efficiency outcomes were commonly reported for documentation and communication tasks. LLM-assisted drafting was associated with perceived reductions in writing time and improved readability in simulated documentation workflows (26, 59). Most published efficiency estimates were derived from controlled tasks rather than prospective studies; efficiency gains can also be offset by verification overhead (5, 6, 60).

### 4.4 User experience, trust, and adoption signals

User experience was often measured through surveys, usability studies, or qualitative interviews. Trust is not equivalent to correctness: systems that produce fluent text may elicit over-reliance, increasing automation-bias risk (7). Adoption-related indicators include acceptance rates, override rates, edit distance, and escalation frequency.

### 4.5 Safety outcomes and clinical risk

Direct safety outcomes were rarely reported. Safety was typically assessed indirectly through expert identification of harmful errors, simulation of high-risk scenarios, or analysis of known failure modes (5, 6, 47). Where safety was assessed, studies emphasised human oversight, escalation mechanisms, and auditability (7, 47, 48, 60).

### 4.6 Equity and subgroup performance

Equity-related outcomes were inconsistently evaluated. Evidence indicates that LLM outputs may reflect biases present in training corpora and may generate disparate responses across demographic contexts (35, 36, 39, 41, 65). Notably, only 23% (*n* = 18) of included studies reported any bias analysis, and only 7% (*n* = 5) conducted predefined subgroup audits. Geographic disparity in study origin (§3.2) compounds demographic underreporting: models validated only on North American or East Asian data may transfer poorly to underrepresented populations (40).

### 4.7 Implications for digital health evaluation

For digital health translation, outcome assessment should be framed as socio-technical evaluation rather than isolated model scoring. A pragmatic evaluation strategy combines: (i) benchmark and robustness testing, (ii) expert review focused on clinically meaningful error types, (iii) workflow metrics that capture net efficiency and revision burden, and (iv) governance-aligned monitoring plans (logging, incident review, drift detection) (7, 15–17, 69).

The current evidence base supports cautious interpretation of reported benefits and highlights the need for prospective studies that connect model-assisted workflows to clinical endpoints and system-level outcomes (76).

Table 9, operationalizes the multidimensional framework by linking application domains to measurable indicators that can be assessed during both pre-deployment validation and post-deployment monitoring. Metrics should be selected based on deployment context, risk tier, and regulatory environment.

**Table 9:** Recommended outcome metrics for LLM evaluation by application domain.

Application Domain	Performance & Correctness	Safety & Risk Indicators	Workflow & Efficiency	Equity & Governance
Clinical documentation & summarisation	Expert-rated factual accuracy; completeness; omission rate; structured data extraction accuracy	Hallucination frequency; critical omission detection; contraindication errors	Net time saved (drafting−review time); edit distance; clinician acceptance rate	Subgroup consistency in summaries; audit logs; version traceability
Clinical decision support & reasoning assistance	Diagnostic suggestion agreement; calibration under uncertainty; robustness to prompt variation	High-risk scenario simulation; incorrect recommendation rate; escalation frequency	Decision review time; override rate; integration latency	Subgroup performance analysis; documented oversight roles; incident reporting mechanisms
Patient communication & health-literacy support	Readability (grade level); clarity; medical correctness; alignment with guidelines	Inappropriate advice detection; risk framing errors; misinformation rate	Response time; clinician revision burden; patient satisfaction scores	Tone/sentiment consistency across demographics; language accessibility; bias audit documentation
Biomedical research & knowledge synthesis	Citation accuracy; summarisation fidelity; retrieval precision/recall	Fabricated-citation rate; synthesis-inaccuracy rate; source misattribution	Literature-screening time saved; reviewer verification burden	Source-diversity auditing; provenance documentation; verification-workflow compliance
Administrative & operational tasks	Coding accuracy; billing consistency; task classification accuracy	Error propagation rate; compliance deviations	Throughput change; processing time reduction; cost proxy indicators	Data governance compliance; logging and auditability; policy alignment

## 5 Discussion

### 5.1 Principal findings

This scoping review of 78 studies identifies five healthcare application domains for LLMs and four outcome categories along which evaluations cluster. The evidence base is rapidly expanding but translationally immature: 89% (*n* = 69) of studies were benchmark- or simulation-based, only 11% (*n* = 9) reported real-world clinical integration, and only 7% (*n* = 5) conducted predefined subgroup audits. Benefits are concentrated in documentation efficiency, text quality, and knowledge synthesis; safety-relevant risks (hallucination, omission, demographic bias, automation bias) persist as recurring properties of probabilistic systems embedded in complex socio-technical environments.

### 5.2 A multidimensional evaluation and governance framework

The five dimensions were derived through a combined inductive–deductive process. Inductively, dimensions 1–4 correspond directly to the four outcome categories synthesised from the 78 included studies (§4): task performance/correctness informed Dimension 1; efficiency and workflow impact informed Dimension 4; user experience and trust informed a sub-component of Dimension 4; and safety, equity, and risk outcomes informed Dimensions 2 and 3. Dimension 5 (governance, transparency, and lifecycle monitoring) arose from a consistent cross-domain finding: the near-universal absence of post-deployment oversight, incident reporting, and model-update documentation. Deductively, each dimension was cross-checked against and refined in relation to MedHELM (27), QUEST (24), ASTRID (29), CONSORT-AI (15), SPIRIT-AI (17), and DECIDE-AI (16) to ensure non-duplication and to define the incremental scope. The three-tier risk model was derived deductively from the EU AI Act's risk-classification logic (70) and the FDA's evolving SaMD guidance, calibrated against the deployment-setting distribution of the reviewed corpus (67% benchmark; 22% simulated; 11% real-world clinical integration). Table 10 (added in the revised manuscript) provides a full cross-mapping of dimensions, extracted themes, and existing frameworks, demonstrating that while MedHELM and ASTRID address Dimensions 1–2, QUEST addresses human-evaluation workflow within Dimension 2, and CONSORT-AI/SPIRIT-AI/DECIDE-AI address reporting transparency within Dimension 5, no existing framework systematically integrates Dimension 3 (equity/fairness audit) and Dimension 4 (workflow integration/human factors) with lifecycle governance across pre- and post-deployment phases.

**Table 10:** Cross-mapping of the five framework dimensions against inductive evidence base and existing evaluation frameworks.

Dimension	Inductive basis (outcome categories from 78 studies)	MedHELM (27)	QUEST (24)	ASTRID (29)	CONSORT-AI (15)	SPIRIT-AI (17)	DECIDE-AI (16)	Coverage gap
D1 — Technical performance & robustness	Task performance & correctness (§4.1); calibration; hallucination taxonomy (§4.2)	✓ Core	Partial	✓ Core	Partial	Partial	Partial	Robustness/adversarial stress-testing not fully covered by any single framework
D2 — Clinical validity & harm modelling	Factuality, hallucination & error modes (§4.2); safety outcomes & clinical risk (§4.5)	✓ Core	✓ Human eval	✓ Core	Partial	Partial	Partial	Structured harm modelling and contraindication auditing absent from existing frameworks
D3 — Equity, fairness & subgroup auditing	Equity & subgroup performance (§4.6); demographic bias; geographic disparity (§3.2)	—	—	—	—	—	—	No existing framework mandates predefined, multi-stratum subgroup auditing
D4 — Workflow integration & human factors	Efficiency & workflow impact (§4.3); user experience, trust & automation bias (§4.4)	—	—	—	—	—	—	No existing framework covers net time saved, override rates, EHR latency, or cognitive workload
D5 — Governance, transparency & lifecycle monitoring	Accountability & oversight gaps identified across all five application domains	—	—	Partial	✓ Reporting	✓ Reporting	✓ Reporting	Lifecycle governance (drift detection, incident reporting, update transparency) not fully covered by any framework

✓=substantially addressed; Partial=partially addressed; —=not addressed. MedHELM, medical holistic evaluation of language models (27); QUEST, quality evaluation of scientific trustworthiness (24); ASTRID, AI safety and transparency framework (29); CONSORT-AI (15), SPIRIT-AI (17), DECIDE-AI (16)=AI reporting extensions.

We propose a five-dimensional evaluation and governance framework operationalised across pre-deployment and post-deployment phases. The framework integrates technical, clinical, equity, workflow, and governance dimensions within a lifecycle perspective and is intended to be operationalised by both researchers (when designing evaluation studies) and healthcare organisations (when assessing deployment readiness).

Dimension 1 — Technical performance and robustness. Task-specific correctness, robustness to prompt variation, sensitivity to incomplete or ambiguous inputs, and reproducibility across repeated runs. Benchmark comparisons may provide baseline context but should be supplemented with stress testing and adversarial scenario simulation (6). Robustness assessment should also evaluate calibration under uncertainty.
Dimension 2 — Clinical validity and harm modelling. Clinical validity requires alignment with accepted standards of care and contextual appropriateness. Evaluation should include structured expert-panel review focusing on clinically meaningful error categories, such as contraindication omission, misinterpretation of temporal relationships, or inappropriate risk stratification.
Dimension 3 — Equity, fairness, and subgroup auditing. Equity assessment should be predefined rather than *post hoc*. Subgroup analyses should include demographic stratification (age, sex, race/ethnicity, language) and contextual factors (e.g., resource-limited settings) (7, 36, 65).
Dimension 4 — Workflow integration and human factors. Workflow evaluation examines how LLM outputs are incorporated into routine practice. Metrics include net time saved, edit distance, override frequency, cognitive workload, and integration latency within EHR systems (7).
Dimension 5 — Governance, transparency, and lifecycle monitoring. Core elements include model cards (71), dataset-provenance transparency, version control, logging of prompts and outputs, and defined oversight roles. Evaluation should differentiate between pre-deployment phases [technical validation, bias audit, safety stress testing, privacy-impact assessment (78)] and post-deployment phases (continuous monitoring, performance-drift detection, incident reporting, periodic bias reassessment, update transparency).

The five dimensions were derived through a combined inductive–deductive process. Inductively, Dimensions 1–4 correspond to the four outcome categories synthesised from the 78 included studies (§4); Dimension 5 arose from consistent findings of accountability and oversight gaps across all application domains. Deductively, each dimension was cross-checked against MedHELM (27), QUEST (24), ASTRID (29), CONSORT-AI (15), SPIRIT-AI (17), and DECIDE-AI (16) to ensure the proposed framework adds scope rather than duplicating established efforts. Table 10 provides the full cross-mapping.

### 5.3 Risk-tiered evaluation and deployment gating

Not all use cases warrant identical evaluative thresholds. A risk-tier model can align evaluation intensity with potential harm:

Tier 1 (low risk): administrative drafting or internal summarisation; emphasis on efficiency and error containment. Minimum standards include general GMLP principles and NIST AI RMF Govern/Map functions.

Tier 2 (moderate risk): clinician-facing documentation or decision-support drafts requiring mandatory human sign-off. Minimum standards include ISO 14971 risk management, IEC 62304 software lifecycle, and GMLP; pre-market notification (SaMD pathway) may apply depending on jurisdiction. *Given that the current evidence base for Tier 2 use cases is predominantly simulation-derived, the following requirements are proposed as minimum standards informed by regulatory guidance, pending prospective validation in real-world clinical settings*.

Tier 3 (higher risk): systems influencing diagnostic or therapeutic decisions; require comprehensive robustness testing, harm modelling, subgroup auditing, and continuous surveillance. *Given that 89% (n = 69) of the reviewed corpus is benchmark- or simulation-derived, these requirements are proposed as minimum standards informed by regulatory guidance and risk-management principles, pending prospective real-world validation.* Minimum pre-deployment standards for Tier 3 include: (i) explicit uncertainty quantification or calibrated confidence reporting; (ii) defined abstention mechanisms for out-of-distribution or high-ambiguity inputs; (iii) mandatory human review and documented sign-off for all outputs that directly influence diagnostic or treatment decisions; and (iv) documented incident-reporting pathways with defined escalation thresholds and root-cause analysis protocols. Tier 3 systems meeting the EU AI Act definition of high-risk AI should demonstrate conformity with ISO 14971, IEC 62304, ISO/IEC 23894, and ISO/IEC 42001, and should implement FDA PCCP and GMLP frameworks for continuous monitoring (see Table 11 for full per-tier regulatory mapping).

**Table 11:** Per-tier mapping of the proposed risk-tier model to regulatory and standards frameworks.

Risk Tier	FDA (US)	ISO/IEC Standards	NIST AI RMF 1.0	EU AI Act
Tier 1 (Low risk) Administrative drafting; internal summarisation	GMLP general principles	ISO 14,971 (risk management — basic)	Gover*n* + Map functions	Minimal-risk provisions (Recital 43)
Tier 2 (Moderate risk) Clinician-facing documentation; decision-support drafts with mandatory human sign-off	GMLP + SaMD guidance; pre-market notification pathway	ISO 14,971 + IEC 62,304 (software lifecycle)	Govern + Map + Measure functions	Art. 9–15 (limited-risk; transparency obligations)
Tier 3 (High risk) Systems influencing diagnostic or therapeutic decisions	PCCP + SaMD; GMLP with continuous monitoring; 510(k)/*de novo* where applicable	ISO 14971 + IEC 62304 + ISO/IEC 23894 (AI risk mgt) + ISO/IEC 42001 (AI MS)	Full RMF cycle: Govern, Map, Measure, Manage	Art. 9–17 (high-risk AI); CE marking; post-market surveillance

GMLP, good machine learning practice; PCCP, predetermined change control plan; SaMD, software as a medical device; RMF, risk management framework; AI MS, ai management system. ISO 14971: risk management for medical devices. IEC 62304: medical device software lifecycle. ISO/IEC 23894: AI risk management. ISO/IEC 42001: AI management systems.

Risk-tiered gating supports proportional regulation and pragmatic innovation while maintaining patient safety, and aligns with the risk-classification logic of the EU AI Act (70) and the FDA's evolving guidance on generative AI in medical software. Table 11 provides explicit per-tier mapping to FDA guidance (GMLP/PCCP/SaMD), ISO standards (14971/IEC 62304/ISO-IEC 23894/42001), NIST AI RMF 1.0, and EU AI Act provisions, to support real-world adoption by hospital procurement teams, software developers, and regulatory affairs professionals.

Table 11 maps each risk tier to accepted regulatory and standards frameworks. For Tier 3 systems meeting the EU AI Act definition of high-risk AI in medical devices, conformity assessment should demonstrate alignment with ISO 14971 (risk management), IEC 62304 (software lifecycle), ISO/IEC 23894 (AI-specific risk management), and ISO/IEC 42001 (AI management systems). In the US context, sponsors should engage with FDA's PCCP and GMLP frameworks at the pre-submission stage. NIST AI RMF 1.0 provides a jurisdiction-agnostic governance scaffold applicable across all tiers.

### 5.4 Toward standardised reporting and comparative evaluation

For the field to mature, evaluation must become comparable across studies. Standardised reporting of model version, prompt structure, deployment context, subgroup analyses, and oversight mechanisms is essential (15–17). Harmonisation with emerging AI-reporting extensions (CONSORT-AI, SPIRIT-AI, DECIDE-AI) and with field-specific frameworks such as MedHELM (27), QUEST (24), ASTRID (29), and LExT (80) can facilitate reproducibility and evidence synthesis. The proposed framework is complementary to these efforts: MedHELM and ASTRID specify benchmark structure and evaluation triads; QUEST specifies human-evaluation workflow; the present framework integrates these with equity (Dimension 3), workflow integration (Dimension 4), and lifecycle-governance dimensions (Dimension 5) — dimensions not systematically addressed by any single existing framework.

### 5.5 Comparison with prior reviews

This review extends prior scoping and systematic syntheses (9–14, 79) in three respects. First, it integrates application domains, outcome categories, evaluation methodologies, and governance considerations within a single analytic structure. Second, it addresses an explicit gap identified by Shool et al. (11) and Artsi et al. (12) — the absence of a multidimensional, lifecycle-aware framework — by operationalising five dimensions across pre- and post-deployment phases. Third, it foregrounds equity not only as a fairness concern but also as a property of the evidence base itself, by reporting the geographic and demographic distribution of included studies.

### 5.6 Implications for healthcare organisations and policy

For healthcare institutions considering LLM adoption, five principles follow from the synthesis: (i) align evaluation intensity with the clinical risk profile of the use case; (ii) include robustness testing, harm modelling, subgroup audits, privacy assessment, and workflow simulation in pre-deployment validation; (iii) require explicit human review and sign-off in moderate- and high-risk contexts; (iv) implement logging, drift detection, incident reporting, and periodic reassessment following model updates; and (v) document subgroup performance and governance mechanisms in alignment with emerging AI-reporting standards (15–17).

## 6 Strengths and limitations

### 6.1 Strengths

Strengths of this review include (i) prospective protocol registration; (ii) reporting against the PRISMA-ScR checklist; (iii) a peer-reviewed search strategy spanning seven bibliographic databases plus preprint and grey-literature sources; (iv) duplicate independent screening and extraction with reported inter-rater agreement; (v) a reflexive thematic-synthesis approach with an explicit reflexivity statement; and (vi) integration of equity reporting into both the analytic framework and the descriptive synthesis.

### 6.2 Limitations

Several limitations should be acknowledged. The field is evolving rapidly; new model releases, new evaluation frameworks, and new regulatory guidance may have appeared between search execution and publication. Heterogeneity of evaluation metrics across included studies precluded any quantitative pooling. The evidence base is dominated by proprietary GPT-class APIs (63%; *n* = 49); findings may transfer imperfectly to open-weight or domain-specialised models. Geographic concentration in North America and East Asia (78% of studies) limits external validity for low- and middle-income contexts (40).

Language restriction to English, Portuguese, and Spanish may have excluded relevant evaluations published in Mandarin, Korean, or other non-Latin scripts. This restriction potentially underrepresents research from East Asian, Middle Eastern, and South Asian institutions and is acknowledged as a source of selection bias.

Consistent with JBI guidance, formal critical appraisal of individual studies was not performed. The proposed framework reflects the disciplinary perspective of the review team and would benefit from co-design with clinicians, patients, and policy-makers in subsequent work.

## 7 Conclusions

LLMs demonstrate versatility across clinical documentation, decision support, patient communication, biomedical research, and administrative workflows. However, current evidence remains predominantly benchmark- or simulation-based, with limited prospective workflow-embedded validation. Reported benefits — drafting efficiency, text clarity, knowledge synthesis — must be balanced against persistent safety-relevant risks: hallucination, omission, demographic bias, privacy vulnerabilities, limited explainability, and automation bias.

A five-dimensional, risk-tiered evaluation and governance framework, applied across pre-deployment and post-deployment phases, can support researchers and healthcare organisations in moving from proof of capability to evidence-based deployment. Translational progress will require prospective, workflow-embedded studies; standardised outcome reporting; equity-centred audits as a default; integration of human factors and safety engineering; and adaptive lifecycle governance for updateable systems.

## Data Availability

The datasets presented in this study can be found in online repositories. The names of the repository/repositories and accession number(s) can be found in the article/Supplementary Material.

## Supplementary material

Supplementary File S1 PRISMA-ScR checklist.

Supplementary File S2 Full search strings (final executed versions, including MeSH/Emtree terms added post-PRESS review); PRESS-2015 peer-review log; corrected citation-chasing dates (1-6 April 2026); PRISMA flow numbers.

Supplementary File S3 Data-extraction template, codebook, and thematic-coding decisions.

Supplementary File S4 Complete list of 78 included studies: study ID, citation, revised five-domain taxonomy assignment, model type, evaluation design, deployment setting, geographic origin, and governance-coding flags. Includes redistribution rationale for Education/training and Research/administrative studies.
