# Supplementary material for: Large language models in healthcare: applications, evaluation frameworks, and governance pathways — a scoping review and multidimensional framework
Source: Front Digit Health. 2026 Sep 18;8:1865568. doi: 10.3389/fdgth.2026.1865568 (PMC13632479; doi:10.3389/fdgth.2026.1865568)
Supplement: Supplementary file 1 [file Datasheet1.pdf]

Supplementary File S1 — PRISMA-ScR Checklist

Companion to "Large Language Models in Healthcare: Applications, Evaluation Frameworks, and Governance Pathways — A Scoping Review and Multidimensional Framework"

This file reports compliance with the Preferred Reporting Items for Systematic Reviews and Meta-Analyses extension for Scoping Reviews (PRISMA-ScR; Tricco et al. 2018, Ann Intern Med 169:467–73, doi:10.7326/M18-0850). The 22 checklist items are reported in the official order; for each item we indicate the section(s) of the main manuscript where the item is reported and the page/section reference. Where an item was deemed not applicable, a justification is provided.

Checklist

Item #	Checklist item	Reported (Y/N/NA)	Manuscript section / page
**1**	Title — Identify the report as a scoping review.	Y	Title page; subtitle includes "A Scoping Review and Multidimensional Framework".
**2**	Structured summary — Provide a structured summary that includes (as applicable): background, objectives, eligibility criteria, sources of evidence, charting methods, results, and conclusions that relate to the review questions and objectives.	Y	Abstract (Background / Objective / Methods / Results / Conclusions / Keywords).
**3**	Rationale — Describe the rationale for the review in the context of what is already known.	Y	§1 Introduction (paragraphs 1–4); contrast with prior reviews [41–43,48,52,67].
**4**	Objectives — Provide an explicit statement of the questions and objectives.	Y	§1 Introduction; five explicit research questions (RQ1–RQ5).
**5**	Protocol and registration — Indicate whether a review protocol exists; state if and where it can be accessed and registration information.	Y	§2.1 Protocol and registration — prospectively registered on the Open Science Framework (OSF DOI to be inserted on acceptance).
**6**	Eligibility criteria — Specify characteristics of the sources of evidence used as eligibility criteria, with rationale.	Y	§2.3 Eligibility criteria; rationale provided for English/Portuguese/Spanish language pragmatism.
**7**	Information sources — Describe all information sources in the search and	Y	§2.4 Information sources and search strategy: PubMed/MEDLINE,

	date last searched.		Embase, Scopus, Web of Science, IEEE Xplore, ACM Digital Library, ACL Anthology, medRxiv/bioRxiv, plus grey-literature scan; dates 1 Jan 2019 – 30 Apr 2026; full execution dates in Supplementary File S2.
**8**	Search — Present the full electronic search strategy for at least one database, including any limits used, such that it could be repeated.	Y	§2.4 (core query); full database-specific strings in Supplementary File S2.
**9**	Selection of sources of evidence — State the process for selecting sources of evidence (i.e., screening and eligibility) included in the scoping review.	Y	§2.5 Study selection — Covidence; pilot screening of 100 records; dual independent title/abstract and full-text screening; Cohen's κ = 0.78; third-reviewer adjudication.
**10**	Data charting process — Describe the methods of charting data from the included sources of evidence (e.g., calibrated forms or forms that have been tested by the team before their use, and whether data charting was done independently or in duplicate) and any processes for obtaining and confirming data from investigators.	Y	§2.6 Data extraction; piloted form, dual independent extraction with consensus; full template in Supplementary File S3.
**11**	Data items — List and define all variables for which data were sought and any assumptions and simplifications made.	Y	§2.6 (variable list) and full codebook in Supplementary File S3.
**12**	Critical appraisal of individual sources of evidence — If done, provide a rationale for conducting it; describe the methods used and how this information was used in any data synthesis.	Y	§2.7 Critical appraisal — formal critical appraisal not undertaken in line with JBI guidance for scoping reviews; methodological-quality flags reported in Table 1 (deployment setting, evaluation design, equity reporting, oversight reporting).
**13**	Synthesis of results — Describe the methods of handling and summarising the data that were charted.	Y	§2.8 Synthesis — Braun and Clarke six-phase reflexive thematic analysis; collaborative codebook; weekly consensus

			meetings.
**14**	Selection of sources of evidence — Give numbers of sources of evidence screened, assessed for eligibility, and included in the review, with reasons for exclusions at each stage, ideally with a flow diagram.	Y	§3.1 Study selection; PRISMA-ScR flow diagram (Figure 1); detailed numbers in Supplementary File S2.
**15**	Characteristics of sources of evidence — For each source of evidence, present characteristics for which data were charted and provide the citations.	Y	§3.2 Characteristics of included studies; Table 1 (n=78); Tables 2–8 organise representative references thematically; full inclusion list in Supplementary File S3.
**16**	Critical appraisal within sources of evidence — If done, present data on critical appraisal of included sources of evidence (see item 12).	NA	Not done; methodological-quality flags reported in Table 1 in lieu of formal appraisal (see item 12 above).
**17**	Results of individual sources of evidence — For each included source of evidence, present the relevant data that were charted that relate to the review questions and objectives.	Y	§3.3 Application domains, §4 Reported outcomes, Tables 2–8; representative findings cited inline against each thematic claim.
**18**	Synthesis of results — Summarise and/or present the charting results as they relate to the review questions and objectives.	Y	§3.3 (RQ1), §4.1–4.7 (RQ2–RQ3), §5.1 (principal findings).
**19**	Summary of evidence — Summarise the main results (including an overview of concepts, themes, and types of evidence available); link to the review questions and objectives, and consider the relevance to key groups.	Y	§5.1 Principal findings; §5.5 Comparison with prior reviews; §5.6 Implications for healthcare organisations and policy.
**20**	Limitations — Discuss the limitations of the scoping review process.	Y	§6 Strengths and Limitations (§6.1 Strengths; §6.2 Limitations) — dedicated section covering rapid field evolution, metric heterogeneity, GPT-class dominance, geographic/language restrictions, absence of formal critical appraisal,

			and reflexive limits of the framework.
**21**	Conclusions — Provide a general interpretation of the results with respect to the review questions and objectives, as well as potential implications and/or next steps.	Y	§7 Conclusions; framework + risk-tier summary; research and policy agenda for translational progress.
**22**	Funding — Describe sources of funding for the included sources of evidence, as well as sources of funding for the scoping review. Describe the role of the funders of the scoping review.	Y	Title-page Funding statement (FCT projects UID/6486/2025, UID/PRR/6486/2025, UID/PRR2/06486/2025); Conflicts-of-interest statement: none declared; funders had no role in design, conduct, analysis, interpretation, or reporting.

Notes on partial-compliance items

Item 12 / Item 16 — Critical appraisal of individual sources of evidence was deliberately omitted, in line with JBI methodological guidance for scoping reviews (Peters et al., JBI Evid Synth 2020;18:2119–26). The review's objective is conceptual mapping and framework construction, not effect estimation or evidence-strength quantification. Four methodological-quality flags (deployment setting, evaluation design, equity reporting, oversight reporting) were nevertheless captured during data extraction and are reported in Table 1 of the main manuscript so that readers can gauge evidence maturity at a glance.

Item 22 — The funders (FCT — Fundação para a Ciência e a Tecnologia, I.P.) had no role in the design or conduct of the review, the collection, analysis, or interpretation of data, the writing of the report, or the decision to submit for publication.

Reference

Tricco AC, Lillie E, Zarin W, O'Brien KK, Colquhoun H, Levac D, Moher D, Peters MDJ, Horsley T, Weeks L, Hempel S, Akl EA, Chang C, McGowan J, Stewart L, Hartling L, Aldcroft A, Wilson MG, Garritty C, Lewin S, Godfrey CM, Macdonald MT, Langlois EV, Soares-Weiser K, Moriarty J, Clifford T, Tunçalp Ö, Straus SE. PRISMA Extension for Scoping Reviews (PRISMA-ScR): Checklist and Explanation. Ann Intern Med. 2018;169(7):467–73. doi:10.7326/M18-0850.
