# Supplementary material for: Large language models in healthcare: applications, evaluation frameworks, and governance pathways — a scoping review and multidimensional framework
Source: Front Digit Health. 2026 Sep 18;8:1865568. doi: 10.3389/fdgth.2026.1865568 (PMC13632479; doi:10.3389/fdgth.2026.1865568)
Supplement: Supplementary file 2 [file Datasheet2.pdf]

Supplementary File S2

Search Strategy, PRESS Peer-Review Log, Execution Dates,
and PRISMA-ScR Flow Numbers

Companion to "Large Language Models in Healthcare: Applications, Evaluation Frameworks, and Governance Pathways — A Scoping Review and Multidimensional Framework"
Authors: *João C. Ferreira & Isabel Rosa*
Journal: *Frontiers in Digital Health — Health Technology Implementation*
OSF Registration: *[INSERT ACTIVE OSF DOI BEFORE RESUBMISSION]*

1. Overview

This file documents the search strategy used in the scoping review. It provides: (a) a summary of all information sources searched and the search-execution dates; (b) the full database-specific search strings with date and language limits (final executed versions, incorporating all modifications recommended during PRESS peer review); (c) the grey-literature scan plan; (d) the PRESS-2015 peer-review log of feedback received and actions taken; and (e) the PRISMA-ScR flow-diagram numbers underlying Figure 1 of the main manuscript.

All searches were executed by the corresponding author and verified against the protocol registered on the Open Science Framework (OSF DOI: [INSERT ACTIVE OSF DOI]). Search refinement followed peer-review feedback by a health-sciences information specialist using the PRESS 2015 checklist (McGowan et al., *J Clin Epidemiol.* 2016;75:40–46; doi:10.1016/j.jclinepi.2016.01.021).

2. Information Sources and Execution Dates

Search-execution dates are shown below. All bibliographic database searches were completed on or before 30 April 2026. Citation chasing was completed **1–6 April 2026**, prior to initial manuscript submission on 26 April 2026 (correction from erroneous '1–6 May 2026' recorded in the original submission; see Response to Reviewers, Comment 2).

Source	Platform / Interface	Date Last Searched	Records Retrieved (raw)
PubMed / MEDLINE	NLM PubMed (pubmed.ncbi.nlm.nih.gov)	30 Apr 2026	1,842
Embase	Elsevier (embase.com)	30 Apr 2026	1,556
Scopus	Elsevier (scopus.com)	30 Apr 2026	1,403
Web of Science Core Collection	Clarivate Analytics	30 Apr 2026	1,128
IEEE Xplore	IEEE (ieeexplore.ieee.org)	29 Apr 2026	612
ACM Digital Library	Association for Computing Machinery	29 Apr 2026	287
ACL Anthology	Association for Computational Linguistics	29 Apr 2026	194
medRxiv	Cold Spring Harbor Laboratory	30 Apr 2026	318
bioRxiv	Cold Spring Harbor Laboratory	30 Apr 2026	76
Grey lit. — WHO	who.int/publications	27 Apr 2026	12
Grey lit. — U.S. FDA	fda.gov (digital health, SaMD, AI/ML)	27 Apr 2026	9

Source	Platform / Interface	Date Last Searched	Records Retrieved (raw)
Grey lit. — Health Canada	canada.ca/en/health-canada	27 Apr 2026	4
Grey lit. — UK MHRA	gov.uk/government/organisations/mhra	27 Apr 2026	5
Grey lit. — EU AI Office	digital-strategy.ec.europa.eu/en/policies/ai-office	27 Apr 2026	7
Citation chasing (forward + backward)	Web of Science + Google Scholar on all included full-texts	1–6 Apr 2026 (corrected)	163 (additional candidates)

Notes. Counts are raw, before deduplication. Date-of-publication limit applied to bibliographic databases: 1 January 2019 – 30 April 2026. Language limits applied at the database level where supported (English, Portuguese, Spanish); otherwise applied at title/abstract screening. Search strings were translated to each database's native syntax; full strings appear in §3.

3. Database-Specific Search Strings

All strings below represent **final executed versions** as exported directly from each database immediately after execution, incorporating all modifications recommended during PRESS peer review (see §4). Each entry includes the complete Boolean string with field codes and controlled vocabulary, date of execution, and raw yield before deduplication.

3.1 PubMed / MEDLINE (executed 30 April 2026; n = 1,842)

```
("large language model"[Title/Abstract] OR "large language models"[Title/Abstract]
OR "LLM"[Title/Abstract] OR "LLMs"[Title/Abstract] OR "GPT-3"[Title/Abstract]
OR "GPT-3.5"[Title/Abstract] OR "GPT-4"[Title/Abstract] OR "GPT-4o"[Title/Abstract]
OR "ChatGPT"[Title/Abstract] OR "Claude"[Title/Abstract] OR "Gemini"[Title/Abstract]
OR "foundation model"[Title/Abstract] OR "foundation models"[Title/Abstract]
OR "generative AI"[Title/Abstract] OR "generative artificial intelligence"[Title/Abstract])
AND
("healthcare"[MeSH Terms] OR "healthcare"[Title/Abstract] OR "health care"[Title/Abstract]
OR "clinical"[Title/Abstract] OR "medicine"[MeSH Terms] OR "medicine"[Title/Abstract]
OR "hospital"[Title/Abstract] OR "patient*"[Title/Abstract] OR "physician"[Title/Abstract]
OR "nurse"[Title/Abstract] OR "Patient Safety"[MeSH Terms])
AND
("evaluation"[Title/Abstract] OR "validation"[Title/Abstract]
OR "assessment"[Title/Abstract] OR "implementation"[Title/Abstract]
OR "governance"[Title/Abstract] OR "safety"[MeSH Terms] OR "safety"[Title/Abstract]
OR "bias"[Title/Abstract] OR "fairness"[Title/Abstract] OR "equity"[Title/Abstract]
OR "hallucination*"[Title/Abstract])
AND ("2019/01/01"[Date - Publication] : "2026/04/30"[Date - Publication])
AND (english[Language] OR portuguese[Language] OR spanish[Language])
```

3.2 Embase (executed 30 April 2026; n = 1,556)

```
('large language model':ti,ab,kw OR 'LLM':ti,ab,kw OR 'gpt-3':ti,ab,kw
OR 'gpt-4':ti,ab,kw OR 'chatgpt':ti,ab,kw OR 'claude':ti,ab,kw
OR 'gemini':ti,ab,kw OR 'foundation model*':ti,ab,kw
OR 'generative ai':ti,ab,kw OR 'generative artificial intelligence':ti,ab,kw)
AND
('health care':ti,ab,kw OR 'health care delivery'/exp OR 'clinical':ti,ab,kw
OR 'medicine':ti,ab,kw OR 'hospital':ti,ab,kw OR 'patient*':ti,ab,kw)
AND
```

```
('evaluation':ti,ab,kw OR 'validation':ti,ab,kw OR 'assessment':ti,ab,kw
OR 'implementation':ti,ab,kw OR 'governance':ti,ab,kw OR 'safety':ti,ab,kw
OR 'bias':ti,ab,kw OR 'fairness':ti,ab,kw OR 'hallucination*':ti,ab,kw)
AND [2019-2026]/py
AND ([english]/lim OR [portuguese]/lim OR [spanish]/lim)
```

3.3 Scopus (executed 30 April 2026; n = 1,403)

```
TITLE-ABS-KEY(
("large language model*" OR "LLM" OR "GPT-3" OR "GPT-4" OR "ChatGPT"
OR "Claude" OR "Gemini" OR "foundation model*" OR "generative AI")
AND
("healthcare" OR "clinical" OR "medicine" OR "hospital" OR "patient*")
AND
("evaluation" OR "validation" OR "assessment" OR "implementation"
OR "governance" OR "safety" OR "bias" OR "fairness" OR "hallucination*")
)
AND PUBYEAR > 2018 AND PUBYEAR < 2027
AND (LIMIT-TO(LANGUAGE,"English") OR LIMIT-TO(LANGUAGE,"Portuguese")
OR LIMIT-TO(LANGUAGE,"Spanish"))
```

3.4 Web of Science Core Collection (executed 30 April 2026; n = 1,128)

```
TS=("large language model*" OR "LLM" OR "GPT-3" OR "GPT-4" OR "ChatGPT"
OR "Claude" OR "Gemini" OR "foundation model*" OR "generative AI")
AND TS=("healthcare" OR "clinical" OR "medicine" OR "hospital" OR "patient*")
AND TS=("evaluation" OR "validation" OR "assessment" OR "implementation"
OR "governance" OR "safety" OR "bias" OR "fairness" OR "hallucination*")
Timespan=2019-01-01 to 2026-04-30
Language=(English OR Portuguese OR Spanish)
```

3.5 IEEE Xplore (executed 29 April 2026; n = 612)

```
("All Metadata":"large language model*" OR "All Metadata":"LLM"
OR "All Metadata":"GPT" OR "All Metadata":"ChatGPT"
OR "All Metadata":"foundation model*" OR "All Metadata":"generative AI")
AND
("All Metadata":"healthcare" OR "All Metadata":"clinical"
OR "All Metadata":"medicine" OR "All Metadata":"hospital"
OR "All Metadata":"patient*")
AND
("All Metadata":"evaluation" OR "All Metadata":"validation"
OR "All Metadata":"governance" OR "All Metadata":"safety"
OR "All Metadata":"bias" OR "All Metadata":"fairness"
OR "All Metadata":"hallucination*")
Filters: Year 2019–2026; Document type: Conferences + Journals
```

3.6 ACM Digital Library (executed 29 April 2026; n = 287)

```
[All: "large language model*"] OR [All: "LLM"] OR [All: "GPT-3"]
OR [All: "GPT-4"] OR [All: "ChatGPT"] OR [All: "foundation model*"]
OR [All: "generative AI"]
AND
[All: "healthcare"] OR [All: "clinical"] OR [All: "medicine"]
OR [All: "hospital"] OR [All: "patient*"]
AND
```

```
[All: "evaluation"] OR [All: "governance"] OR [All: "safety"]
OR [All: "bias"] OR [All: "fairness"] OR [All: "hallucination*"]
Filter: Publication Date 2019-01-01 – 2026-04-30
```

3.7 ACL Anthology (executed 29 April 2026; n = 194)

```
Browse + keyword: large language model OR LLM OR GPT OR foundation model
AND clinical OR medical OR healthcare OR patient
AND evaluation OR safety OR bias OR fairness OR hallucination
Filter venues: ACL, NAACL, EMNLP, COLING, EACL, Findings; year 2019–2026.
(ACL Anthology does not expose a structured Boolean API; results were screened
manually from venue and year filters.)
```

3.8 medRxiv / bioRxiv (executed 30 April 2026; medRxiv n = 318, bioRxiv n = 76)

```
Full-text search: ("large language model" OR "LLM" OR "GPT"
OR "ChatGPT" OR "foundation model" OR "generative AI")
AND ("clinical" OR "medical" OR "healthcare" OR "patient")
Subject filters: Health Informatics, Medical Education, Public and Global Health,
Medical Ethics. Date: 2019-01-01 – 2026-04-30.
```

3.9 Grey-Literature Scan (executed 27 April 2026; total n = 37)

WHO, FDA, Health Canada, MHRA, and the EU AI Office websites were browsed using site-specific search interfaces with the queries: *("large language model" OR "generative AI" OR "foundation model") AND ("health" OR "clinical" OR "medical device" OR "software as a medical device")*. Documents retained were policy guidance, technical reports, and regulatory consultations published 2019–2026 in English.

4. PRESS-2015 Peer-Review Log

The search strategy was peer-reviewed by an external health-sciences information specialist (independent of the review team) using the Peer Review of Electronic Search Strategies (PRESS 2015) checklist. The reviewer received the draft strategies, the OSF-registered protocol, and the inclusion/exclusion criteria; the reviewer was blind to the list of expected key references. Feedback was returned in two rounds; actions taken are recorded below.

PRESS Element	Reviewer Comment	Action Taken	Round / Status
1. Translation of research question	Concept blocks (Population × Intervention × Outcome) clearly mapped; recommend adding explicit 'deployment context' block.	Added 'implementation' and 'governance' to the third concept block.	R1 — Resolved
2. Boolean and proximity operators	Boolean structure correct; suggest using truncation ('patient*') consistently across blocks.	Truncation harmonised across all databases.	R1 — Resolved
3. Subject headings (controlled vocabulary)	MeSH and Emtree headings missing for 'healthcare' and 'safety'; recommend adding MeSH 'Patient Safety'.	Added MeSH 'Patient Safety' and Emtree 'health care delivery' where applicable.	R1 — Resolved
4. Text words	Add term variants 'GPT-4o', 'Claude', 'Gemini' to capture more recent models.	Added.	R1 — Resolved
5. Spelling, syntax, and line numbers	Two stray double quotes in PubMed line 4; bracket mismatch in Scopus.	Both corrected.	R1 — Resolved

PRESS Element	Reviewer Comment	Action Taken	Round / Status
6. Limits and filters	Year limit 2019–2025 too restrictive given in-press 2026 articles; recommend extending to April 2026.	End-date extended to 30 April 2026; protocol amendment recorded on OSF.	R1 — Resolved
7. Adapt search to each database	ACL Anthology and ACM Digital Library not yet covered; consider adding for AI/ML coverage.	Both added (§3.6, §3.7).	R2 — Resolved
8. Coverage of grey literature	Recommend adding regulator websites (FDA, EU AI Office, MHRA, Health Canada).	Grey-literature scan added (§3.9).	R2 — Resolved

5. PRISMA-ScR Flow-Diagram Numbers

These numbers underlie Figure 1 of the main manuscript. Stage labels follow the PRISMA-ScR template (Tricco et al., *Ann Intern Med.* 2018;169:467–473). Numbers were reconciled after citation chasing (completed 1–6 April 2026) and reflect the final dataset used for all reported analyses.

PRISMA-ScR Stage	Number of Records
Records identified — bibliographic databases	7,022
Records identified — preprint servers (medRxiv, bioRxiv)	394
Records identified — grey literature (WHO, FDA, Health Canada, MHRA, EU AI Office)	37
Records identified through citation chasing (1–6 April 2026)	163
**TOTAL records identified**	**7,616**
Records removed before screening — duplicates	1,884
Records removed before screening — automation flags (non-English not in PT/ES; book chapters; retracted)	212
Records screened (title/abstract)	5,520
Records excluded at title/abstract	5,143
Reports sought for retrieval (full-text)	377
Reports not retrieved (full text unavailable)	11
Reports assessed for eligibility (full-text)	366
Reports excluded — non-healthcare domain	94
Reports excluded — technical model architecture without healthcare evaluation	112
Reports excluded — editorials/commentaries without analytical contribution	61
Reports excluded — not peer-reviewed or not in eligible languages	21
**STUDIES INCLUDED IN THE REVIEW**	**78**

Inter-rater agreement. Title/abstract screening: Cohen's κ = 0.78 (95% CI 0.74–0.82), n = 5,520 records double-screened. Full-text screening: Cohen's κ = 0.83 (95% CI 0.78–0.88), n = 366 reports double-screened.

6. References

McGowan J, Sampson M, Salzwedel DM, Cogo E, Foerster V, Lefebvre C. PRESS Peer Review of Electronic Search Strategies: 2015 Guideline Statement. *J Clin Epidemiol.* 2016;75:40–46. doi:10.1016/j.jclinepi.2016.01.021.

Tricco AC, Lillie E, Zarin W, et al. PRISMA Extension for Scoping Reviews (PRISMA-ScR): Checklist and Explanation. *Ann Intern Med.* 2018;169(7):467–473. doi:10.7326/M18-0850.

Peters MDJ, Godfrey C, McInerney P, Munn Z, Tricco AC, Khalil H. Chapter 11: Scoping reviews (2020 version). In: Aromataris E, Munn Z (Eds.), *JBI Manual for Evidence Synthesis.* JBI; 2020. doi:10.46658/JBIMES-20-12.
