# Supplementary material for: Large language models in healthcare: applications, evaluation frameworks, and governance pathways — a scoping review and multidimensional framework
Source: Front Digit Health. 2026 Sep 18;8:1865568. doi: 10.3389/fdgth.2026.1865568 (PMC13632479; doi:10.3389/fdgth.2026.1865568)
Supplement: Supplementary file 3 [file Datasheet3.pdf]

Supplementary File S3 — Data-Extraction Template, Codebook, and Thematic-Coding Decisions

Companion to "Large Language Models in Healthcare: Applications, Evaluation Frameworks, and Governance Pathways — A Scoping Review and Multidimensional Framework".

Part A — Extraction workflow

Two reviewers extracted data independently from each included study using the structured template specified in Part B. Extraction was performed in Covidence (Veritas Health Innovation, Melbourne) with a custom field set mirroring Part B. The form was piloted on ten randomly selected studies and refined; two fields (model release date; abstention behaviour) were added during piloting. Discrepancies between reviewers were reconciled by consensus; unresolved discrepancies (n = 4 across the full sample) were referred to a third reviewer for adjudication.

Part B — Data-extraction template

All variables extracted from each included study, with definitions, field types, and (where relevant) controlled-vocabulary value sets. Variables in bold are required; non-bold variables are conditional or optional.

Field / Section	Type	Definition / value set
**Bibliographic**		
**Study ID**	Integer	Sequential ID assigned at import; primary key.
**First author surname**	Text	Surname of first author as printed.
**Year of publication**	Integer	Calendar year of formal publication; for in-press records, year of advance online publication.
**Country of corresponding author**	Text	Country listed in corresponding-author affiliation.
**Journal / venue**	Text	Full journal title or conference name.
**DOI / persistent identifier**	Text	DOI; arXiv/medRxiv identifier accepted for preprint-only records.
**Funding source**	Text	As reported by the study; verbatim.
**Population and setting**		
**Clinical setting**	Controlled	Inpatient | Outpatient | Emergency | Primary care | Pharmacy | Mixed | Not specified.
**Specialty / domain**	Text	Free text (e.g., radiology, psychiatry, paediatrics).
**Patient population (when applicable)**	Text	Age band, sex distribution, condition; 'N/A — synthetic data' permitted.
**Sample size (clinical)**	Integer	Number of patients/encounters (when applicable).

**Model characteristics**		
**Model family**	Controlled	GPT-3 | GPT-3.5 | GPT-4 | GPT-4o | Claude (any) | Gemini (any) | LLaMA (any) | Mistral (any) | PaLM/MedPaLM | Domain fine-tuned | Other (specify).
**Model version / release**	Text	Specific version string (e.g., gpt-4-0613); release date if reported.
**Access mode**	Controlled	Proprietary API | Open weights / open source | Domain fine-tuned (specify base) | Mixed.
**Multimodal**	Controlled	Yes (vision) | Yes (audio) | Yes (other) | No.
**RAG / retrieval-augmented**	Controlled	Yes — closed corpus | Yes — open web | Yes — clinical knowledge base | No | Not reported.
**Prompting strategy**	Controlled	Zero-shot | Few-shot | Chain-of-thought | Tool-use / agentic | Mixed | Not reported.
**Deployment and evaluation design**		
**Deployment setting**	Controlled	Benchmark / retrospective only | Simulated workflow | Real-world clinical integration.
**Evaluation design**	Controlled	Cross-sectional benchmarking | Retrospective chart comparison | Prospective observational | Controlled trial | Mixed methods.
**Comparator**	Controlled	Human (clinician) | Other AI system | None | Pre/post (within-subject) | Multiple.
**Sample size (evaluation)**	Integer	Number of cases, items, or queries evaluated.
**Reproducibility statement**	Controlled	Code released | Prompts released | Both | Neither | Partially.
**Outcomes reported**		
**Task performance metrics**	Multi-select	Accuracy | F1 | AUROC | BLEU | ROUGE | BERTScore | Expert rubric | Pairwise preference | Other (specify).
**Calibration / abstention reported?**	Yes/No/NS	Whether the study reported calibration of confidence or abstention behaviour.
**Hallucination / factuality assessed?**	Yes/No/NS	Whether factuality was explicitly evaluated.
**Hallucination type characterised**	Multi-select	Intrinsic | Extrinsic | Misattribution | Omission | None.
**Efficiency outcomes**	Multi-select	Drafting time | Review time | Net time saved | Edit distance | Throughput | Cognitive load | Burnout proxy | None.
**User-experience outcomes**	Multi-select	Acceptability | Usability score | Trust | Satisfaction | Override / acceptance

		rate | None.
**Safety outcomes**	Multi-select	Adverse-event rate | Critical-omission rate | Wrong-dose rate | Inappropriate advice rate | Escalation frequency | Simulated harm | None.
**Equity and bias reporting**		
**Subgroup analyses prespecified?**	Yes/No/NS	Whether subgroup analyses were declared a priori.
**Demographic stratification reported**	Multi-select	Age | Sex / gender | Race / ethnicity | Language | Insurance / SES | Geographic | None reported.
**Bias-mitigation strategy applied**	Multi-select	Prompt adjustment | Fine-tuning | Dataset augmentation | Output filtering | RLHF / RLAIF | None | Other.
**Governance and oversight**		
**Privacy / data-handling described**	Yes/No/NS	Whether de-identification, hosting, and consent are reported.
**Human oversight specified**	Controlled	Mandatory sign-off | Optional review | Background only | None | Not specified.
**Post-deployment monitoring described**	Yes/No/NS	Whether logging, drift detection, or audit cadence is described.
**Reporting standard followed**	Multi-select	CONSORT-AI | SPIRIT-AI | DECIDE-AI | TRIPOD+AI | None reported.
**Conflict-of-interest disclosure**	Text	Verbatim from the publication.
**Methodological-quality flags (Table 1, manuscript)**		
**Deployment maturity flag**	Tier	Tier A: real-world integration; Tier B: simulated workflow; Tier C: benchmark only.
**Equity-reporting flag**	Tier	Tier A: prespecified subgroup audit; Tier B: post-hoc subgroup analysis; Tier C: none.
**Oversight-reporting flag**	Tier	Tier A: mandatory sign-off + monitoring; Tier B: oversight specified; Tier C: not specified.
**Reproducibility flag**	Tier	Tier A: code + prompts + data released; Tier B: partial release; Tier C: none.

Part C — Thematic-analysis codebook

The codebook below was developed iteratively during the six phases of Braun and Clarke's reflexive thematic analysis (familiarisation; coding; theme generation; reviewing; defining; writing). Codes are organised under the five overarching themes that ultimately structure §3 (Application domains), §4 (Reported outcomes), and §5 (Discussion / Framework) of the main manuscript.

Theme 1: Application domain (RQ1)

Code	Code label	Definition / inclusion rule
**APP-DOC**	Clinical documentation and summarisation	Note drafting, discharge summaries, structured extraction from EHR free text, dialogue summarisation.
**APP-CDS**	Clinical decision support and reasoning	Differential diagnosis, triage suggestion, clinical question answering, clinical-pathway-aware reasoning.
**APP-PAT**	Patient communication and education	In-basket replies, lay-language translation, education content, conversational chatbots facing patients.
**APP-RES**	Biomedical research and knowledge synthesis	Literature screening, summarisation of biomedical literature, hypothesis generation.
**APP-ADM**	Administrative and operational	Coding/billing assistance, scheduling, throughput optimisation, regulatory paperwork.

Theme 2: Outcome domain (RQ2)

Code	Code label	Definition / inclusion rule
**OUT-PERF**	Task performance and correctness	Benchmark accuracy, expert rubric ratings, calibration, abstention behaviour.
**OUT-EFF**	Efficiency and workflow effects	Time saved, edit distance, throughput, cognitive load, revision burden.
**OUT-UX**	User experience, trust, and adoption	Acceptability, perceived usefulness, override and acceptance rates, automation bias signals.
**OUT-SAF**	Safety, equity, and risk-related outcomes	Hallucination, omission, contraindication errors, subgroup disparity, simulated harm.

Theme 3: Evaluation methodology (RQ3)

Code	Code label	Definition / inclusion rule
**EVAL-BENCH**	Benchmark / retrospective evaluation	Cross-sectional benchmarking, exam-style questions, retrospective chart comparison.
**EVAL-SIM**	Simulated workflow evaluation	Mock EHR, structured simulation studies, role-play.
**EVAL-PROSP**	Prospective workflow-embedded evaluation	Pragmatic, hybrid effectiveness–implementation, stepped-wedge.
**EVAL-EXP**	Expert / human evaluation	Panel review, rubric, pairwise comparison, qualitative interviews.
**EVAL-STRESS**	Stress / robustness testing	Prompt perturbation, adversarial ambiguity, jailbreak, distribution

		shift.

Theme 4: Risk and governance (RQ4)

Code	Code label	Definition / inclusion rule
**RISK-HALL**	Hallucination and factual error	Intrinsic, extrinsic, misattribution, omission; clinically consequential vs. inconsequential.
**RISK-BIAS**	Demographic and contextual bias	Race, sex/gender, age, language, SES, geographic; differential tone/framing.
**RISK-PRIV**	Privacy and data-handling	De-identification, data residency, model-input retention, BAA/DPA.
**RISK-AUTO**	Automation bias and over-reliance	Passive acceptance, attentional disengagement, deskilling.
**GOV-DOC**	Documentation and transparency	Model cards, dataset provenance, version control, prompt logging.
**GOV-OVER**	Oversight roles and accountability	Sign-off requirements, escalation, incident reporting.
**GOV-LIFE**	Lifecycle monitoring	Drift detection, periodic reassessment, update transparency.

Theme 5: Framework constructs (RQ5)

Code	Code label	Definition / inclusion rule
**FW-D1**	Dimension 1 — Technical performance and robustness	Correctness, robustness, calibration, reproducibility, abstention.
**FW-D2**	Dimension 2 — Clinical validity and harm modelling	Standard-of-care alignment, harm modelling, scenario simulation.
**FW-D3**	Dimension 3 — Equity, fairness, and subgroup auditing	Predefined subgroup audits, qualitative bias dimensions.
**FW-D4**	Dimension 4 — Workflow integration and human factors	Net time saved, edit distance, override frequency, cognitive load, usability testing.
**FW-D5**	Dimension 5 — Governance, transparency, and lifecycle monitoring	Pre-deployment validation, post-deployment monitoring, institutional QA integration.
**FW-RISK**	Risk tier (1–3)	Tier 1 administrative; Tier 2 clinician-facing with sign-off; Tier 3 decision-influencing.

Part D — Thematic-coding decisions

The decisions below capture the most consequential analytic choices made during synthesis. They are reported here in the spirit of reflexive transparency.

D1. Treating ‘benchmark / retrospective’ as a single deployment category.

Studies that used closed-question benchmarks (e.g., USMLE-style items) and studies that used retrospective chart comparison were combined into a single deployment-maturity tier. Although the underlying data sources differ, both

share the property that no live patient or clinician is exposed to the model output during the evaluation, and reviewers therefore judged them comparable for the purpose of judging translational maturity.

D2. Including studies that used multimodal models when language was a core component.

Pure imaging-classification systems (no language input/output) were excluded; multimodal studies in which the model produced or consumed natural-language reports (e.g., chest-X-ray report generation) were retained. This decision is documented in the Methods section (§2.3) and reflects the protocol.

D3. Treating 'omission of clinically critical information' as a hallucination subtype.

We followed Ji et al. (2023) in their broad framing of hallucination but emphasised omission in the medical-context taxonomy because clinically harmful errors frequently take the form of missing information rather than fabricated information. This decision is reflected in §4.2 and in the codebook (RISK-HALL).

D4. Distinguishing 'bias analysis reported' from 'subgroup audit prespecified'.

Of 78 included studies, 18 (23%) reported some form of bias analysis, but only six (7%) had prespecified the subgroup audit in the protocol. We coded these as separate fields in extraction (see Part B) and report both numbers in §3.2 and §4.6 to avoid overstating the maturity of equity reporting.

D5. Mapping outcomes to Table 9 (recommended metrics).

Outcomes were grouped under the four columns of Table 9 by consensus rather than by counting: a metric was assigned to 'safety & risk' if its primary use in the study was to detect or prevent harm, even when the same metric (e.g., recall) might also appear under 'performance & correctness'. This judgemental mapping is acknowledged as a reflexive choice.

D6. Adjudication of borderline inclusions.

Four studies were borderline for inclusion: two evaluated non-clinical text (e.g., medical school application essays), one evaluated lay health-information retrieval, and one evaluated a veterinary use case. The two essay studies and the veterinary study were excluded; the lay-health-information study was included on the basis of its explicit framing as a patient-facing tool. The third reviewer adjudicated three of the four cases.

D7. Theme saturation.

We assessed thematic saturation informally. By study 60, no new first-order codes were emerging; further studies refined existing codes rather than producing new ones. Saturation was therefore judged sufficient at n = 78.

D8. Treatment of in-press / preprint records.

Preprints were retained only if they had subsequently been accepted for publication in a peer-reviewed venue with a DOI assigned at the time of writing. This is more restrictive than many scoping reviews; we judged the trade-off reasonable in a fast-moving field where unpublished claims may be revised.

Part E — Suggested reporting templates for future studies

To support standardised reporting (manuscript §5.4), we provide three minimal templates that future LLM-in-healthcare studies could adopt. Each template is keyed to one or more dimensions of the proposed framework.

E.1 Pre-deployment evaluation report (Dimensions 1–3 + 5)

Element	Required content
**Model identity**	Family, version, release date, access mode.
**Population**	Population definition; subgroup strata prespecified.
**Use case**	Task definition, risk tier (1–3), expected operating mode.
**Technical evaluation**	Benchmark suite, robustness tests, calibration, abstention.

**Clinical validity**	Standard-of-care alignment, harm modelling scenarios.
**Equity audit**	Predefined subgroup analyses, qualitative dimensions.
**Privacy / governance**	DPIA summary, model card, oversight roles.
**Decision**	Approve / approve-with-conditions / decline; conditions stated.

E.2 Workflow-embedded study report (Dimension 4)

Element	Required content
**Setting**	Site type, EHR, integration architecture, inclusion period.
**Comparators**	Pre/post, parallel, hybrid effectiveness–implementation, etc.
**Workflow metrics**	Net time saved, edit distance, override rate, escalation frequency.
**Human-factors metrics**	Cognitive load, trust calibration, automation-bias signals.
**Adverse events**	Pre-defined; severity grading scheme stated.
**Cost / resource use**	Compute, latency, energy where measurable.

E.3 Post-deployment monitoring plan (Dimension 5)

Element	Required content
**Monitoring cadence**	Frequency of automated and manual reviews.
**Drift indicators**	Distributional drift, performance drift, complaint rate.
**Incident reporting**	Trigger thresholds, owner, escalation chain.
**Update transparency**	Notification policy, re-validation triggers.
**Bias reassessment**	Cadence and trigger conditions.
**Sunset criteria**	Conditions under which the system would be withdrawn.

References

Braun V, Clarke V. Using thematic analysis in psychology. Qual Res Psychol. 2006;3(2):77–101. doi:10.1191/1478088706qp063oa.

Ji Z, Lee N, Frieske R, et al. Survey of hallucination in natural language generation. ACM Comput Surv. 2023. doi:10.1145/3571730.

Peters MDJ, Marnie C, Tricco AC, et al. Updated methodological guidance for the conduct of scoping reviews. JBI Evid Synth. 2020;18(10):2119–2126. doi:10.11124/JBIES-20-00167.
